# Influence of Nutrition and Lifestyle on Bone Mineral Density in Children From Adoptive and Biological Families

**DOI:** 10.2188/jea.JE20130094

**Published:** 2014-05-05

**Authors:** Selma Cvijetic, Irena Colic Baric, Zvonimir Satalic, Irena Keser, Jasminka Bobic

**Affiliations:** 1 Institute for Medical Research and Occupational Health, Zagreb, Croatia; 2 Laboratory for Nutrition Science, Faculty of Food Technology and Biotechnology, University of Zagreb, Zagreb, Croatia

**Keywords:** bone mineral density, adopted children, lifestyle, heredity, nutrition

## Abstract

**Background:**

The precise contributions of hereditary and environmental factors to bone density are not known. We compared lifestyle predictors of bone density among adopted and biological children.

**Methods:**

The study comprised 18 adopted children (mean [SD] age, 14.0 [4.1] years) with their non-biological parents and 17 children with their biological parents. Bone mineral density (BMD; g/cm^2^) was measured at the lumbar spine, total femur, and distal radius. Nutritional intake was assessed by food frequency questionnaire. Information on smoking and physical activity was obtained by questionnaire.

**Results:**

Intakes of all nutrients, corrected for energy intake, and all lifestyle characteristics except sleep duration were similar in biological children and their parents. As compared with their parents, adopted children had significantly different energy, protein, and calcium intakes and physical activity levels. In a regression model, BMD z scores of adopted children and their parents were significantly inversely associated at the spine and total femur, whereas BMD z scores of biological children and their parents were significantly positively associated at all measurement sites. The greatest proportion of total variance in BMD was accounted for by calcium intake among adopted children and by parental BMD among biological children.

**Conclusions:**

For some lifestyle characteristics and nutrient intakes, the differences between parents and children were more obvious among adoptive families than among biological families. The most important lifestyle predictor of bone density was calcium intake.

## INTRODUCTION

Bone is living tissue that changes constantly: parts of old bone are removed and replaced by new bone. During childhood and adolescence, much more bone is deposited than withdrawn, so the skeleton grows in size and density. Depending on skeletal site, up to 90% of peak bone mass is acquired by age 18 years, and bone mass continues to increase until around age 30.^1^^–^^4^ Peak bone mass is influenced by a variety of genetic and environmental factors. Genetic factors may account for up to 75% of bone mass, while environmental factors such as diet and exercise habits account for the remaining 25%.^5^^–^^9^

Since peak bone density is predominantly formed during adolescence, when children live with their parents, family lifestyle habits may have an important role in the environmental contribution to peak bone density. Family lifestyle refers to the way that families live and coexist together on a daily basis, the way a family eats, the amount of exercise they get, and all other habits and patterns that parents and children have as individuals and as part of a family.

Parents usually have considerable influence over the types and amount of food made available to their children.^10^ Research shows that a child’s preference for certain foods depends on the food’s availability in the home.^11^ For example, daughters’ milk consumption correlated with their mothers’ lifelong milk and calcium consumption.^12^ Parents also serve as role models for other aspects of healthy living. The balance between parenting rules and habits and a child’s responsiveness is most important for a healthful lifestyle in a family.^13^ In addition to the family, lifestyle habits regarding diet and activity are reinforced by friends, schools, and community resources in a child’s environment.

Adopted children, especially those who were adopted in early childhood, mostly accept the lifestyle habits in the family environment. However, some children might have negative affects or emotional withdrawal from the adoptive family, especially during adolescence, when they experience identity development.^14^ There are wide individual differences in such reactions; however, negative adolescent behavior can also be present in biological families.

We analyzed the similarity and relationships of several lifestyle habits with bone mineral density (BMD) in adopted children and their non-biological parents and compared those findings with data from children who lived with their biological parents. We sought to determine whether the impact of lifestyle factors on bone density differed between children who live with their adoptive parents and those who live with biological parents. We hypothesized that lifestyle habits would be similar between children and their parents, both in adoptive and biological families, and that the impact of lifestyle factors on BMD would be similar in children living with adoptive or biological parents, since lifestyle habits are modeled in families. We also attempted to identify the lifestyle factors that had the greatest influence on BMD.

## METHODS

### Subjects

#### Recruitment of adopted children

To recruit adopted children, we contacted the Center for Social Care, which is responsible for children without parental care and for the adoption process. The center has access to the names and addresses of families with adopted children. Each county in Croatia has 1 center for social care, and we contacted the Center for Social Care in Zagreb city. Before contacting the center, we requested permission for this study from the Ministry of Health and Social Care, which has jurisdiction over all Centers for Social Care in the country. The Ministry delivered written approval allowing the Center for Social Work to contact families with adopted children, to request their participation in this study. Initial contact with families was by phone and by the officer from the Center for Social Care, who briefly informed the parents of the study aims and design. If parents agreed to participate in the study, we were allowed to obtain their names, phone numbers, and addresses. Recruitment was limited to children who were adopted during early childhood, ie, during the first 3 years of life. A total of 23 families were contacted, 6 of which declined to participate; 4 families could not be contacted. Ultimately, data from 18 children (10 boys, 8 girls) with 26 parents were analyzed.

#### Recruitment of biological children

Biological children were recruited from the database of another study, which aimed to determine the impacts of heredity and environment on peak BMD in young men and women. That study was conducted at Zagreb University.^15^^,^^16^ We selected 17 students (10 girls, 7 boys; 4 students had brothers or sisters) and their parents (13 mothers, 13 fathers).

Adopted and biological children who had medical conditions, or used drugs, known to affect bone density (eg, hyperthyroidism; renal, hepatic, and gonadal dysfunction; malignancies; malabsorption; corticosteroids; anticoagulants; and antiepileptic drugs) were excluded from the study. These exclusion criteria were not applied to the parents. There were 2 mothers receiving thyroid hormone therapy, 1 mother with type 1 diabetes, and 1 father with type 2 diabetes. None of the mothers or daughters from adoptive families used contraceptives. In the biological families, 5 daughters and 2 mothers used contraceptives.

#### Standard protocol approvals, registrations, and patient consent

Before inclusion in the study, informed consent was obtained from all participants. The study was approved by the Ethics Committee of the Institute for Medical Research and Occupational Health.

#### Anthropometry and bone density measurement

Height and weight were measured using a portable stadiometer and electronic scale. Body mass index (BMI) was calculated as weight (kg) divided by height (m^2^).

BMD (g/cm^2^) was measured using dual energy X-ray absorptiometry (Lunar-Prodigy, Madison, WI, USA). Measurements were made in the lumbar spine (L2–L4), proximal femur, and distal third of the radius. The in vivo coefficient of variation was 1.5% for the lumbar spine, 2.1% for the femur, and 2.2% for the distal radius. BMD was expressed as z score, which represents the number of standard deviations with respect to the age-matched mean BMD, as indicated by the manufacturer’s reference values.

#### Diet assessment

A food frequency questionnaire was used for dietary assessment and was administered in the form of a personal interview by trained personnel.^17^ The reference period was the previous year, and each subject was asked to recall consumption frequency ranging from once a month to once or more a day. Portion size was defined using life-sized food photographs in which a small, medium, and large portion of each food was shown.^18^ Data were converted to average daily nutrient intakes using food composition tables. In Croatia, there is only 1 food composition database available.^19^ Since this database contains a limited number of food items, US Department of Agriculture food composition tables were also used.^20^ Calcium and vitamin D intakes were compared with recently published estimated average requirements (EARs).^21^ Micronutrient intake was evaluated based on the principle of nutritive density, which is micronutrient intake expressed per energy unit (1000 kcal) using cut-off values derived from dietary reference intake values.^22^

#### Lifestyle assessment

Information on lifestyle habits, including smoking, sleep duration, and physical activity, were obtained using an interviewer-administered questionnaire designed for our previous study of BMD.^15^

Physical activity was recorded by quantifying sports and work activities: duration (years) and frequency (hours per week) of sports activity and intensity (moderate/hard) and frequency (hours per week) of other physical activities. The frequency of sports activities and other moderate and/or hard physical activities were categorized and scored as: 1) never, 2) 0.5 to 1 hour/week, 3) 2 to 3 hours/week, 4) 4 to 6 hours/week, 5) 7 to 10 hours/week, 6) 11 to 20 hours/week, 7) 21 to 30 hours/week, and 8) more than 31 hours/week. The score for sports activity was calculated by multiplying duration and frequency (1 to 8) of sports activity. The final score for physical activity was calculated for each subject by summing the sports activity score with the frequency of moderate and hard work activity score. Those subjects with a higher final score were judged to be more physically active.

The smoking index was calculated by multiplying number of cigarettes per day by years of smoking.

#### Statistics

Data were analyzed using the software Statistica, version 10.0 (StatSoft Inc., Tulsa, OK, USA). The results are shown as mean (SD) and ranges. Differences between groups (means) were tested using the *t*-test. The relation between 2 variables was evaluated by Pearson correlation analysis. A multiple regression model was created with the BMD z score of the child as a dependent variable. Independent variables included parent BMD z score, calcium intake, physical activity, age, and BMI. BMD z scores were used for both correlation and regression analyses. Mid-parent values, ie, the average of values for the father and mother, were used for all variables in both regression and correlation analyses of the association between parents’ and children’s lifestyle characteristics and BMD. The distribution of variables was tested using the Kolmogorov-Smirnov test. Variables that were not distributed normally (protein and carbohydrate intake) were recalculated to new variables, using the logarithmic function.

To determine the percentage of total variation in child BMD that could be explained by predictor variables, we used squared semi-partial correlation, which can be computed in the multiple regression module, ie, the proportion of variance accounted for by the predictors relative to the total variance of the dependent variable. Thus, it is an indicator of the “practical relevance” of the predictor, because it is scaled to the total variability in the dependent variable. In all tests, a *P* value less than 0.05 was considered significant.

## RESULTS

The mean (SD) age of adopted children was 14.0 (4.1) years, and they were significantly younger than the biological children (*P* < 0.001) (Tables 1 and 2). However, no significant differences were found in BMD z scores, BMI, PAI, or any component of nutrition intake between adopted and biological children.

**Table 1.** Age, anthropometry, lifestyle characteristics, bone mineral density, and nutrient intake in adopted children and their parents

Variable	Adopted children (*N* = 18)	Parents (*N* = 26)	*P*^a^
	
	Mean (SD) [range]	Mean (SD) [range]	
Age (years)	14.0 (4.1) [7.9 to 20.0]	49.0 (5.7) [40.0 to 65.0]	<0.001
Height (cm)	157.7 (16.4) [130.0 to 180.0]	167.2 (10.6) [150.0 to 183.0]	0.021
Weight (kg)	50.4 (15.4) [28.0 to 81.0]	80.5 (16.6) [48.0 to 120.0]	<0.001
BMI (kg/m^2^)	19.8 (3.1) [13.2 to 26.6]	28.5 (4.1) [20.7 to 38.3]	<0.001
PAI	4.8 (1.4) [3.1 to 5.8]	4.0 (1.2) [3.5 to 5.5]	n.s.
Smoking index	50 (0.0) [50 to 50] (N 1)	237.5 (152.3) [75 to 525] (N 8)	n.s.
Sleep duration (h)	8.3 (1.9) [5 to 10]	7.1 (1.3) [6 to 10]	0.011
Fractures (No.)	2.0 (1.4) [1 to 3]	1.2 (0.5) [1 to 2]	n.s.
Spine BMD Z score	1.4 (1.1) [−1.4 to 2.8]	−0.1 (0.9) [−2.3 to 1.7]	<0.001
Total femur BMD Z score	0.9 (1.0) [−2.1 to 2.3]	0.5 (0.8) [−1.1 to 2.4]	n.s.
Radius 1/3 BMD Z score	−0.8 (0.9) [−2.4 to 1.5]	−0.0 (0.9) [−1.6 to 2.3]	0.015
Energy (kcal)	3177.1 (1070.0) [1369.1 to 5401.2]	2170.5 (950.3) [833.5 to 4246.5]	0.008
Protein (g/1000 kcal)	33.8 (5.5) [25.8 to 45.6]	39.2 (5.8) [30.4 to 50.4]	0.011
Fat (g/1000 kcal)	37.0 (6.9) [23.2 to 46.0]	36.6 (3.8) [29.8 to 45.2]	n.s.
Carbohydrate (g/1000 kcal)	136.7 (20.1) [106.9 to 174.2]	129.0 (16.6) [81.1 to 156.8]	n.s.
Dietary fiber (g/1000 kcal)	10.1 (3.5) [6.2 to 19.6]	11.3 (2.3) [6.5 to 19.1]	n.s.
Calcium (mg/1000 kcal)	395.3 (99.7) [276.4 to 593.9]	356.8 (118.3) [201.9 to 599.3]	n.s.
Vitamin D (IU/1000 kcal)	27.8 (10.2) [15.3 to 47.4]	33.3 (15.0) [16.1 to 73.9]	n.s.

BMI = body mass index; PAI = physical activity index.

^a^Differences between means were tested with Student *t*-test.

**Table 2.** Age, anthropometry, lifestyle characteristics, bone mineral density, and nutrient intake in biological children and their parents

Variable	Biological children (*N* = 17)	Parents (*N* = 26)	*P*^a^
	
	Mean (SD) [range]	Mean (SD) [range]	
Age (years)	19.7 (2.2) [11.6 to 21.7]	47.1 (3.4) [42.3 to 52.5]	<0.001
Height (cm)	165.7 (6.2) [153.0 to 178.0]	171.0 (9.2) [152.0 to 186.0]	0.044
Weight (kg)	59.5 (11.0) [41.0 to 80.0]	76.1 (12.1) [56.0 to 105.0]	<0.001
BMI (kg/m^2^)	21.5 (3.1) [16.8 to 28.6]	26.1 (2.7) [20.9 to 30.6]	<0.001
PAI	4.1 (1.5) [2.0 to 6.0]	4.3 (1.0) [2.0 to 6.0]	n.s.
Smoking index	241.6 (231.9) [50 to 525] (N 3)	325.0 (86.6) [225 to 375] (N 3)	n.s.
Sleep duration (h)	8.3 (0.7) [7 to 9]	7.0 (0.7) [6 to 8]	<0.001
Fractures (No.)	1.0 (0.0) [1 to 1]	1.0 (0.0) [1 to 1]	/
Spine BMD Z score	0.9 (1.2) [−1.0 to 2.9]	−0.0 (1.1) [−1.7 to 2.4]	0.010
Total femur BMD Z score	1.4 (2.0) [−1.1 to 5.0]	0.5 (0.9) [−1.3 to 2.7]	n.s.
Radius 1/3 BMD Z score	−0.9 (0.9) [−3.1 to 0.5]	−0.6 (0.5) [−1.7 to 0.9]	n.s.
Energy (kcal)	2602.3 (921.5) [1419.3 to 4449.1]	2484.2 (569.6) [1658.8 to 4059.9]	n.s.
Protein (g/1000 kcal)	40.8 (7.7) [26.2 to 51.2]	35.7 (7.2) [19.3 to 47.4]	n.s.
Fat (g/1000 kcal)	36.1 (5.3) [25.3 to 42.6]	35.7 (7.2) [19.3 to 47.4]	n.s.
Carbohydrate (g/1000 kcal)	122.2 (35.2) [14.1 to 163.8]	124.5 (26.6) [89.7 to 192.7]	n.s.
Dietary fiber (g/1000 kcal)	10.5 (4.2) [6.8 to 20.9]	11.2 (4.7) [7.3 to 24.0]	n.s.
Calcium (mg/1000 kcal)	477.5 (132.3) [327.3 to 767.1]	457.0 (168.2) [197.5 to 846.8]	n.s.
Vitamin D (IU/1000 kcal)	29.8 (21.0) [8.0 to 93.9]	34.00 (17.6) [3.6 to 56.9]	n.s.

BMI = body mass index; PAI = physical activity index.

^a^Differences between means were tested with Student *t*-test.

Mean calcium intake was in accordance with the EAR in adopted and biological children. Parents of adopted children had a mean (SD) calcium intake of 762.5 (375.6) mg, which is below the age- and sex-specific EAR. The mean intakes of other nutrients in adopted and biological children were greater than EAR. As compared with their parents, adopted children had a significantly higher energy intake (*P* = 0.008) and a significantly lower protein intake corrected for energy intake (*P* = 0.011). There was no significant difference in nutrition intake between biological children and their parents. In a comparison of BMD z scores between adopted children and their parents, spine BMD z score was significantly higher (*P* < 0.001), while the radius BMD score was significantly lower (*P* = 0.015) in adopted children. Spine BMD z score was significantly higher in biological children than in their parents (*P* = 0.010).

The mean (SD) duration of contraceptive use in biological families was 3.1 (2.2) years. There was no significant correlation between duration of contraception and z scores (*r* = 0.20 spine; *r* = 0.51 femur; *r* = 0.06 radius).

In an analysis of differences between children and their mothers and fathers examined separately, the results in biological families showed only a significantly higher BMI in parents (*P* = 0.001 vs mothers; *P* < 0.001 vs fathers) and significantly longer sleep duration in children (*P* = 0.002 vs mothers; *P* = 0.003 vs fathers). In adoptive families, mothers had significantly lower physical activity (*P* = 0.032), and fathers had a significantly lower calcium intake (*P* = 0.001), than their children. Parents significantly varied in height, weight, and BMD z score of the spine and radius, which were predictably higher in men (adoptive families: *P* < 0.001 height, *P* = 0.002 weight, *P* < 0.001 z score spine, *P* = 0.015 z score radius; biological families: *P* < 0.001 height, *P* = 0.001 weight, *P* = 0.026 z score spine). There were no significant differences in BMI, PAI, smoking index, sleep duration, number of fractures, or intakes of any analyzed nutrients between mothers and fathers in adoptive and biological families, with the exception of calcium intake, which was significantly higher in mothers in adoptive families (*P* = 0.001). Correlational analysis showed a tendency toward an inverse correlation in BMD z scores between both adopted and biological children and their parents. No significant correlations were found in the intakes of any analyzed nutrition component or in physical activity between children and parents in either group of subjects. There were significant correlations in calcium intake between adoptive mothers and fathers (*r* = 0.690, *P* = 0.023) and in protein intake (*r* = 0.865, *P* < 0.001) between biological mothers and fathers. There was no significant correlation in BMI, sleep duration, PAI, or smoking index between mothers and fathers in either group. Associations of BMDs at different skeletal sites with potential confounding variables were analyzed by multiple regression (Tables 3 and 4). The number of confounders was limited by the number of subjects. We included in the model age, sex, BMI, PAI, calcium intake, and parent BMD z score as independent variables. When controlling for age, sex, and BMI, the BMD z score of adopted children was significantly associated with PAI (*P* < 0.001 for total femur and radius) and with calcium intake (*P* = 0.001 for spine and *P* < 0.001 for total femur and radius) (Table 3). There was a significant inverse association between adopted children’s and parents’ BMD z scores at the spine (*P* = 0.002) and total femur (*P* < 0.001). In the biological children, children’s BMD z score was significantly associated with PAI (*P* < 0.001 for total femur and radius) and calcium intake (*P* = 0.031 for spine, *P* = 0.006 for total femur, and *P* < 0.001 for radius). A significant positive association was found between children’s and parents’ BMD z score at all measurement sites (*P* < 0.001). After accounting for age, sex, and BMI, the greatest proportion of total variance in BMD z score in adopted children was accounted for by calcium intake (10.8% at the spine, 17.0% at the femur, and 66.1% at the radius; *P* < 0.001). In biological children, total variance in BMD z score was accounted for mostly by parents’ BMD (48.4% at the spine, 21.5% at the total femur; *P* < 0.001), with the exception of the radius, where calcium intake was the most important predictor of total variance in BMD z score (54.4%; *P* < 0.001).

**Table 3.** Association of BMD z score at different skeletal sites (dependent variable) with predictors in adopted children

Predictors	Z score spine		Z score total femur		Z score radius	
		
	b	*P*	b	*P*	b	*P*
Age	−0.068	n.s.	−0.136	<0.001	−0.204	<0.001
Sex	0.079	0.025	−0.013	n.s.	−0.447	<0.001
BMI	0.045	n.s.	0.058	n.s.	0.006	n.s.
PAI	0.039	n.s.	0.172	<0.001	0.226	<0.001
Calcium intake	0.101	0.001	0.183	<0.001	0.562	<0.001
Parent Z score spine	−0.106	0.002				
Parent Z score total femur			−0.208	<0.001		
Parent Z score radius					0.002	n.s.

BMI = body mass index; PAI = physical activity index; b = standardized regression coefficient.

**Table 4.** Association of BMD z score at different skeletal sites (dependent variable) with predictors in biological children

Predictors	Z score spine		Z score total femur		Z score radius	
		
	b	*P*	b	*P*	b	*P*
Age	−0.239	0.004	−0.107	<0.001	0.040	n.s.
Sex	0.182	<0.001	−0.449	<0.001	0.284	<0.001
BMI	0.143	<0.001	0.284	0.032	0.076	0.004
PAI	0.031	n.s.	0.169	<0.001	0.439	<0.001
Calcium intake	0.545	0.031	0.096	0.006	0.601	<0.001
Parent Z score spine	0.328	<0.001				
Parent Z score total femur			0.259	<0.001		
Parent Z score radius					0.210	<0.001

BMI = body mass index; PAI = physical activity index; b = standardized regression coefficient.

## DISCUSSION

By analyzing several lifestyle habits, we found that, as compared with biological children, adopted children differed more prominently from their parents in important components of lifestyle, including nutrition. As expected, absolute energy and nutrient intakes were significantly higher in children than in their parents, since the period of intensive growth is accompanied by higher energy intake in comparison with other age groups.^23^

However, the results of nutrient density analysis did not show significant differences between children and parents, which reveals the expected similarities in food choice within a family ie, there were differences in the quantity of the food, but the quality of the diet, as indicated by nutrient density, was similar. The high similarity in nutrition and lifestyle between mothers and fathers allowed the use of mid-parent values to analyze children/parents associations. However, more differences in lifestyle were found between children and parents in adoptive families when the analysis was performed between children and their mothers and fathers separately. Those findings indicated that, as compared with biological children, adopted children differed more greatly in lifestyle from their parents.

We presume that adoptive parents in this study were making substantial efforts to provide their children with good care and support and adequate nutrition, even though they may have been less conscientious with themselves. Our results were similar to those of the 2003 National Survey of Children’s Health, which enrolled a sample of 102 353 children, including 2903 adopted children, and estimated 31 indicators of health and well-being among adopted and biological children.^24^ The results suggested that adopted children had worse health than biological children but that they usually lived in a supportive environment that ensured good health care quality.^25^ Other studies suggested that parents who adopted children invested more time in their children than did biological parents.^26^^,^^27^ They usually provided better education and financial support to adoptees. It is possible that adoptive parents sometimes invest more in adoptees because such children are more likely than genetic children to need help.

In general, energy and nutrition intakes were high in all groups. All dietary assessment methods are subject to error, such as response errors, which might result in underreporting or under-consumption.^28^^,^^29^ The use of an FFQ could lead to over- or underestimation. To overcome the apparent overestimation of nutrient intakes in this study, energy and nutrient intakes were also presented relative to energy intake, and the results of the FFQ were primarily used for comparison between groups rather than interpreted as true usual intakes.

The BMD of the present biological children, who lived with their biological parents, was significantly and positively influenced by their parents’ BMD. The lack of a relationship between the BMD of adopted children and that of their non-biological parents was confirmed: there was a negative trend in the contribution of non-biological parents’ BMD to the variance of BMD in their adopted children.

As mentioned in our previous study of BMD and heritability,^15^ we hypothesize that lifestyle predictors not considered in this study might have influenced bone density. The number of predictors was limited by the relatively small number of participants in each analyzed group (adopted/biological children/parents). Factors such as low serum vitamin D level, maternal diet, vitamin D deficiency during pregnancy, and low birth size, which were not analyzed in this study, might be important predictors of BMD in young people. Non-genetic variance may have been decreased by the fact that we did not analyze variance separately among female and male students, who differed significantly in some lifestyle characteristics, such as physical activity. Different studies have found different impacts of lifestyle habits on peak bone density. Generally, dietary calcium predicts between 10% to 15% of skeletal calcium retention during adolescence.^30^^,^^31^ Some studies have shown a greater impact on bone density from dietary calcium than from physical activity^32^^,^^33^ while others have found the opposite.^34^ However, most studies agree on the importance of the interaction between mechanical, hormonal, and dietary factors.^35^^,^^36^ We are not aware of any other study of bone density in adopted children. Therefore, we cannot directly compare our results with the findings of studies with similar designs. Most studies of adopted children investigated their psychological status. Very few studies of adopted children have examined their physical health versus that of children who lived with their biological parents.^26^^,^^37^^,^^38^ The numerous family and twin studies of the genetic component of BMD and osteoporosis have found that heredity accounted for 44% to 92% of bone density.^7^^,^^8^^,^^39^^–^^41^ We used a “family design” to investigate the environmental influence on bone density by comparing that influence between children from adoptive and biological families. We found that adopted children and their parents differ more greatly in lifestyle habits than do biological children and their parents. We thus conclude that some lifestyle habits, like nutrition and affinity for physical activity, also have a genetic predisposition.

The most important limitations of this study are that both groups were small and that the group with biological children was not adjusted by age and sex. The period between age 14 and 20 years is crucial for bone mass development since peak bone density is usually obtained by age 20 in most skeletal regions. However, the primary aim of this study was to compare parent–child similarity in bone density development. We were interested in the influence of heredity on bone density and also in the influence of potentially similar lifestyle characteristics in the same family on bone density.

Another limitation of this study is that the physical activity questionnaire was not validated. However, we also used this questionnaire in a previous study of peak BMD^15^ and found a significant difference in physical activity between students from a faculty of kinesiology who were involved in sports activities and students from other faculties. We therefore believe that this questionnaire is an adequate indicator of physical activity.

We conclude that some important lifestyle characteristics, like nutrition, are more likely to differ between children and parents in adoptive families, as compared with biological families. Among lifestyle characteristics, the most important predictor of bone density in adopted children was calcium intake, which was the second most important predictor, after heredity, in children with biological parents.

## References

[1] Lin YC, Lyle RM, Weaver CM, McCabe LD, McCabe GP, Johnston CC, Peak spine and femoral neck bone mass in young women. Bone. 2003;32:546–53 10.1016/S8756-3282(03)00062-0 12753871

[2] Lu PW, Briody JN, Ogle GD, Morley K, Humphries IR, Allen J, Bone mineral density of total body, spine, and femoral neck in children and young adults: a cross-sectional and longitudinal study. J Bone Miner Res. 1994;9:1451–8 10.1002/jbmr.5650090918 7817830

[3] Theintz G, Buchs B, Rizzoli R, Slosman D, Clavien H, Sizonenko PC, Longitudinal monitoring of bone mass accumulation in healthy adolescents: evidence for a marked reduction after 16 years of age at the levels of lumbar spine and femoral neck in female subjects. J Clin Endocrinol Metab. 1992;75:1060–5 1400871 10.1210/jcem.75.4.1400871

[4] Matkovic V, Jelic T, Wardlaw GM, Ilich JZ, Goel PK, Wright JK, Timing of peak bone mass in Caucasian females and its implication for the prevention of osteoporosis. J Clin Invest. 1994;93:799–808 10.1172/JCI117034 8113412 PMC293933

[5] Krall EA, Dawson-Hughes B Heritable and life-style determinants of bone-mineral density. J Bone Miner Res. 1993;8:1–9 10.1002/jbmr.5650080102 8427042

[6] Nordström P, Lorentzon R Influence of heredity and environment on bone density in adolescent boys: A parent-offspring study. Osteoporos Int. 1999;10:271–7 10.1007/s001980050226 10692974

[7] Mitchell BD, Kammerer CM, Schneider JL, Perez R, Bauer RL Genetic and environmental determinants of bone mineral density in Mexican Americans: results from the San Antonio Family Osteoporosis Study. Bone. 2003;33:839–46 10.1016/S8756-3282(03)00246-1 14623060

[8] Jouanny P, Guillemin F, Kuntz C, Jeandel C, Pourel J Environmental and genetic factors affecting bone mass-similarity of bone density among members of healthy families. Arthritis Rheum. 1995;38:61–7 10.1002/art.1780380110 7818574

[9] Wang X, Kammerer CM, Wheeler VW, Patrick AL, Bunker CH, Zmuda JM Genetic and environmental determinants of volumetric and areal BMD in multi-generational families of African ancestry: The Tobago family health study. J Bone Miner Res. 2007;22:527–36 10.1359/jbmr.070106 17227221

[10] Scaglioni S, Arrizza C, Vecchi F, Tedeschi S Determinants of children’s eating behavior. Am J Clin Nutr. 2011;94 Suppl:2006S–11S 10.3945/ajcn.110.001685 22089441

[11] Rhee KE Childhood overweight and the relationship between parent behaviors, parenting style, and family functioning. Ann Am Acad Polit Soc Sci. 2008;615:11–37 10.1177/0002716207308400

[12] Ulrich CM, Georgiou CC, Snow-Harter CM, Gillis DE Bone mineral density in mother-daughter pairs: relations to lifetime exercise, lifetime milk consumption, and calcium supplements. Am J Clin Nutr. 1996;63:72–9 8604673 10.1093/ajcn/63.1.72

[13] Enten RS, Golan M Parenting styles and weight-related symptoms and behaviors with recommendations for practice. Nutr Rev. 2008;66:65–75 10.1111/j.1753-4887.2007.00009.x 18254872

[14] Grotevant HD Family processes, identity development, and behavioral outcomes for adopted adolescents. J Adolesc Res. 1997;12:139–61 10.1177/0743554897121008

[15] Cvijetic S, Colic Baric I, Satalic Z Influence of heredity and environment on peak bone density: A parent-offspring study. J Clin Densitom. 2010;13:301–6 10.1016/j.jocd.2010.03.003 20542459

[16] Cvijetic S, Colic Baric I, Keser I, Rumbak I, Satalic Z Influence of heredity and environment on peak bone density: a review of studies in croatia. Arh Hig Rada Toksikol. 2011;63 Suppl 1:11–5 10.2478/10004-1254-63-2012-2130 22548848

[17] Satalić Z, Colic Baric I, Keser I Diet quality in Croatian university students: Energy, macronutrient and micronutrient intakes according to gender. Int J Food Sci Nutr. 2007;58:398–410 10.1080/09637480701252393 17558731

[18] Hess MA. Portion Photos of Popular Foods. American Dietetic Association and Center for Nutrition Education, University of Wisconsin-Stout, 1.1.1997.

[19] Kaić-Rak A, Antonić K. Tables of contents of food and drinks. Zagreb: Croatian Institute of Public Health; 1990 (in Croatian).

[20] U.S. Department of Agriculture, Agricultural Research Service. USDA National Nutrient Database for Standard Reference, Release 21. 2007. Nutrient Data Laboratory. Available from: http://www.ars.usda.gov/nutrientdata/

[21] Food and Nutrition Board, Institute of Medicine. Dietary Reference Intakes for Calcium, and Vitamin D. Washington, DC: National Academy Press; 2011.

[22] Lee RD, Nieman DC. Nutritional Assessment. 3rd ed. New York: McGraw-Hill Companies; 2003.

[23] Shomaker LB, Tanofsky-Kraff M, Savastano DM, Kozlosky M, Columbo KM, Wolkoff LE, Puberty and observed energy intake: boy, can they eat! Am J Clin Nutr. 2010;92:123–9 10.3945/ajcn.2010.29383 20504975 PMC2884323

[24] Bramlett MD, Foster EB, Frasier AM, Satorius J, Skalland BJ, Nysse-Carris KL, Design and Operation of the National Survey of Adoptive Parents. National Center for Health Statistics. Vital Health Stat 1. 2010;(50):1–154 20737837

[25] Bramlett MD, Radel LF, Blumberg SJ The health and well-being of adopted children. Pediatrics. 2007;119 Suppl 1:S54–60 10.1542/peds.2006-2089I 17272586

[26] Hamilton L Adoptive Parents, Adaptive Parents: Evaluating the Importance of Biological Ties for Parental Investment. Am Sociol Rev. 2007;72:95–116 10.1177/000312240707200105

[27] Gibson K Differential parental investment in families with both adopted and genetic children. Evol Hum Behav. 2009;30:184–9 10.1016/j.evolhumbehav.2009.01.001

[28] Cade JE, Burley VJ, Warm DL, Thompson RL, Margetts BM Food-frequency questionnaires: a review of their design, validation and utilisation. Nutr Res Rev. 2004;17:5–22 10.1079/NRR200370 19079912

[29] Masson LF, McNeill G, Tomany JO, Simpson JA, Peace HS, Wei L, Statistical approaches for assessing the relative validity of a food-frequency questionnaire: use of correlation coefficients and the kappa statistic. Public Health Nutr. 2003;6:313–21 10.1079/PHN2002429 12740081

[30] Weaver CM The role of nutrition on optimizing peak bone mass. Asia Pac J Clin Nutr. 2008;17 Suppl 1:135–7 18296321

[31] Braun M, Palacios C, Wigertz K, Jackman LA, Bryant RJ, McCabe LD, Racial differences in skeletal calcium retention in adolescent girls on a range of controlled calcium intakes. Am J Clin Nutr. 2007;85:1657–63 17556706 10.1093/ajcn/85.6.1657

[32] Iuliano-Burns S, Saxon L, Naughton G, Gibbons K, Bass SL Regional specificity of exercise and calcium during skeletal growth in girls: a randomised controlled trial. J Bone Miner Res. 2003;18:156–62 10.1359/jbmr.2003.18.1.156 12510818

[33] Matkovic V, Fontana D, Tominac C, Goel P, Chesnut CH 3rd Factors that influence peak bone mass formation: a study of calcium balance and the inheritance of bone mass in adolescent females. Am J Clin Nutr. 1990;52:878–88 2239765 10.1093/ajcn/52.5.878

[34] Ruiz JC, Mandel C, Garabedian M Influence of spontaneous calcium intake and physical exercise on the vertebral and femoral bone mineral density of children and adolescents. J Bone Miner Res. 1995;10:675–82 10.1002/jbmr.5650100502 7639101

[35] Borer KT Physical activity in the prevention and amelioration of osteoporosis in women: interaction of mechanical, hormonal and dietary factors. Sports Med. 2005;35:779–830 10.2165/00007256-200535090-00004 16138787

[36] Branca F, Valtueña S Calcium, physical activity and bone health–building bones for a stronger future. Public Health Nutr. 2001;4:117–23 10.1079/PHN2000105 11255501

[37] Roeber BJ, Tober CL, Bolt DM, Pollak SD Gross motor development in children adopted from orphanage settings. Dev Med Child Neurol. 2012;54:527–31 10.1111/j.1469-8749.2012.04257.x 22413752 PMC3439199

[38] Teilmann G, Petersen JH, Gormsen M, Damgaard K, Skakkebaek NE, Jensen TK Early puberty in internationally adopted girls: hormonal and clinical markers of puberty in 276 girls examined biannually over two years. Horm Res. 2009;72:236–46 10.1159/000236085 19786795

[39] Makovey J, Nguyen TV, Naganathan V, Wark JD, Sambrook PN Genetic effects on bone loss in peri- and postmenopausal women: a longitudinal twin study. J Bone Miner Res. 2007;22:1773–80 10.1359/jbmr.070708 17620052

[40] Flicker L, Hopper JL, Rodgers L, Kaymakci B, Green RM, Wark JD Bone density determinants in elderly women: a twin study. J Bone Miner Res. 1995;10:1607–13 10.1002/jbmr.5650101102 8592936

[41] Van Pottelbergh I, Goemaere S, Zmierczak H, De Bacquer D, Kaufman JM Deficient acquisition of bone during maturation underlies idiopathic osteoporosis in men: Evidence from a three-generation family study. J Bone Miner Res. 2003;18:303–11 10.1359/jbmr.2003.18.2.303 12568407

